# Diversity analysis and genome-wide association studies of grain shape and eating quality traits in rice (*Oryza sativa* L.) using DArT markers

**DOI:** 10.1371/journal.pone.0198012

**Published:** 2018-06-01

**Authors:** Maurice Mogga, Julia Sibiya, Hussein Shimelis, Jimmy Lamo, Nasser Yao

**Affiliations:** 1 Ministry of Agriculture and Food Security, Juba, South Sudan; 2 African Centre for Crop Improvement, School of Agricultural Sciences and Agribusiness, University of KwaZulu-Natal, Pietermaritzburg, South Africa; 3 Cereals Program, National Crops Resources Research Institute (NaCRRI), Kampala, Uganda; 4 Biosciences eastern and central Africa-International Livestock Research Institute (BecA-ILRI) Hub, Nairobi, Kenya; Clemson University, UNITED STATES

## Abstract

Microarray-based markers such as Diversity Arrays Technology (DArT) have become the genetic markers of choice for construction of high-density maps, quantitative trait loci (QTL) mapping and genetic diversity analysis based on their efficiency and low cost. More recently, the DArT technology was further developed in combination with high-throughput next-generation sequencing (NGS) technologies to generate the DArTseq platform representing a new sequencing tool of complexity-reduced representations. In this study, we used DArTseq markers to investigate genetic diversity and genome-wide association studies (GWAS) of grain quality traits in rice (*Oryza sativa* L.). The study was performed using 59 rice genotypes with 525 SNPs derived from DArTseq platform. Population structure analysis revealed only two distinct genetic clusters where genotypes were grouped based on environmental adaptation and pedigree information. Analysis of molecular variance indicated a low degree of differentiation among populations suggesting the need for broadening the genetic base of the current germplasm collection. GWAS revealed 22 significant associations between DArTseq-derived SNP markers and rice grain quality traits in the test genotypes. In general, 2 of the 22 significant associations were in chromosomal regions where the QTLs associated with the given traits had previously been reported, the other 20 significant SNP marker loci were indicative of the likelihood discovery of novel alleles associated with rice grain quality traits. DArTseq-derived SNP markers that include SNP12_100006178, SNP13_3052560 and SNP14_3057360 individually co-localised with two functional gene groups that were associated with QTLs for grain width and grain length to width ratio on chromosome 3, indicating trait dependency or pleiotropic-effect loci. This study demonstrated that DArTseq markers were useful genomic resources for genome-wide association studies of rice grain quality traits to accelerate varietal development and release.

## Introduction

Rice (*Oryza sativa* L.) is increasingly becoming a major food crop in sub-Saharan Africa (SSA). Globally, rice is one of the most widely cultivated cereal crops distributed across diverse geographical, ecological and climatic conditions [1,2]. Given the varied adaptations of rice genotypes, several accessions are available with wide phenotypic and genotypic diversity [3]. A great number of these rice accessions, belonging to different sub-species including *indica*, j*aponica* and *javanica*, have been conserved in global gene banks [4]. This is important as a potential source of reservoir genes that could be exploited in crop improvement programs [5, 6]. However, only a slight amount of the available rice genetic resources have been utilized in most rice breeding programs [1], hence a great genetic similarity exists in most commercial rice cultivars given the narrow genetic base [3].

Most rice breeding programs in SSA face the challenge of improving not only the yield potential but also other important grain quality traits such as cooking and processing qualities [1,7,8]. Furthermore, grain quality and in particular cooking and eating quality always represents a major criterion in evaluating rice grain quality [9]. Rice cooking and eating quality is strongly determined by the level of amylose content (AC) [10,11], where high AC in the endosperm is usually associated with dry, fluffy, and separated cooked rice grains, and represents the key determinant of poor cooking and eating quality [12]. In addition, rice grain shape is an important character which subsequently affects cooking quality [13, 14]. Rice grain shape is determined by its three dimensions including, grain length (GL), grain width (GW) and grain length to width ratio (L/W).

The genetic basis of rice grain shape has been well studied [15, 16] and several quantitative trait loci (QTLs) underlying grain shape have been detected and fine mapped [17, 18] using different populations He et al. [19] identified twelve QTLs associated with rice grain size on chromosomes 2, 3, 4, 5, 6, 7 and 11 using recombinant inbred lines (RILs) derived from the cross of Zhenshan 97 x Minghui 63, Zhang et al. [18] detected three QTLs for rice elongation using a doubled haploid (DH) population derived from ZYQ8 x JX17. Furthermore, Shen et al. [20] used the same DH population and identified fourteen QTLs related to cooking traits. Based on a high-density SNP map, Li et al. [21] identified 17 QTLs that were associated with 12 cooking traits using a population of 132 RILs derived from PA64s x 93–1. However, the identified QTLs may not be sufficient to elucidate the genetic basis of rice grain shape. Furthermore, the varied nature of rice grain shape underscores the need for identifying novel QTLs in order to design a breeding strategy for grain shape improvement, and generating rice cultivars with desirable cooking and eating quality traits [9].

In addition, it is essential to broaden the genetic base of rice genotypes by introducing genes from distant or wild relatives with potential for delivering novel genes or quantitative trait loci (QTLs) for important agronomic traits. Furthermore, the magnitude of genetic variability and the extent to which the desirable characters are heritable largely determines the success of any plant breeding program [22]. Consequently, association mapping (AM) based on phenotypic and genotypic data has been critical in identifying molecular markers or QTLs linked to traits of interest and with potential for use in marker-assisted selection (MAS). This has allowed the use of diverse set of germplasm that provides a broader allelic coverage without necessarily developing bi-parental mapping populations [23].

More recently with the advances in next generation sequencing (NGS) technologies, genotyping by sequencing (GBS) has emerged as a promising genomic approach for simultaneous exploration of plant genetic diversity and molecular marker discovery [24,25,26]. Thus, GBS has effectively been used for single-nucleotide polymorphisms (SNP) marker discovery and QTL identification of tightly linked marker-trait associations [27, 28] and in the application of genomic selection of complex traits for crop improvement [29, 30]. The GBS approach is therefore considered an important cost-effective tool for population genetics, QTL discovery, high-resolution mapping and for genomic selection in plant breeding programs [25, 29].

With advances in microarray-based marker technology, Diversity Arrays Technology (DArT) markers have become the genetic markers of choice for construction of high-density maps, mapping quantitative trait loci (QTL) and genetic diversity analysis based on their efficiency and low cost [31]. Additionally, by combining the complexity reduction of the DArT method with high-throughput next-generation sequencing (NGS) technologies, the DArTseq platform was developed signifying a new implementation of sequencing of complexity-reduced representations [14]. Consequently, DArTseq markers based on GBS technology have been successfully applied for linkage mapping, QTL identification in bi-parental mapping population, genome wide association studies (GWAS), genetic diversity, as well as in marker-assisted and genomic selection [32]. Hence, DArTseq has been widely applied [33, 34, 35] and is rapidly gaining popularity as a preferred method of genotyping by sequencing [32]. The objective of this study was to investigate genetic diversity and genome-wide association studies (GWAS) of grain quality traits in a diverse collection of 59 upland and lowland rice (*Oryza sativa* L.) genotypes.

## Materials and methods

### Germplasm and phenotyping

The present study used a collection of 59 rice genotypes, which included 2 popular landraces, 36 upland and 21 lowland rice collections (Table 1). The above introductions were acquired from the National Crops Resources Research Institute (NaCRRI-Uganda), where they are permanently held, while the landraces (LDR) are collections from South Sudan. Therefore, samples were identified as introductions from the International Rice Research Institute (IRRI), Africa Rice Centre (ARC), National Crops Resources Research Institute (NaCRRI-Uganda), International Center for Tropical Agriculture (CIAT), Madagascar (MDG), Tanzania (TZ) and Institut d’Economie Rurale(IER-Mali). This research study was approved and conducted at the Biosciences eastern and central Africa-International Livestock Research Institute (BecA-ILRI) Hub, Nairobi, Kenya. Test materials were assessed for determinants of grain quality (grain shape, amylose content, and alkali spreading value) using dehusked grains. Grain shape was classified on the basis of grain length (GL), grain width (GW) and length to width ratio (L/W), where measurements were read using a vernier calliper as described by Cruz and Khush [36].

**Table 1:** List of rice genotypes used in the study.

Entry No.	Name/pedigree	Ecology	Origin	PID Ɨ	Entry No.	Name/pedigree	Ecology	Origin	PID Ɨ
1	GSR-I-0057	Lowland	ARC	ARC	31	P5 H12	Upland	NaCRRI	UG
2	K 5	Lowland	NaCRRI	UG	32	P24 H10	Upland	NaCRRI	UG
3	WAC116X NERICA 4	Lowland	Mali	IER	33	CT11891-3-3-3-M-1-2-2-M	Upland	CIAT	CIAT
4	NERICA L 19	Lowland	ARC	ARC	34	P5 H6	Upland	NaCRRI	UG
5	K-85	Lowland	NaCRRI	UG	35	ART12-L4P7-21-4-B-3	Upland	ARC	ARC
6	JARIBU	Lowland	Tanzania	TZ	36	ART10-1L15P1-4-3-1	Upland	ARC	ARC
7	TAI	Lowland	IRRI	IRRI	37	ART2-4L3P1-2-1	Upland	ARC	ARC
8	K85-10	Lowland	NaCRRI	UG	38	SCRIDO 06-2-4-3-4-5	Upland	Madagascar	MDG
9	KOMBOKA	Lowland	IRRI	ARC	39	ART3 -8L6P3-2-3-B	Upland	ARC	ARC
10	1052 SUPA LINE	Lowland	IRRI	IRRI	40	P27 H4	Upland	NaCRRI	UG
11	K 38	Lowland	NaCRRI	UG	41	P26 H1	Upland	NaCRRI	UG
12	TXD 306	Lowland	ARC	ARC	42	ART3-7L9P8-3-5-B-B-2	Upland	ARC	ARC
13	WITA 9	Lowland	ARC	ARC	43	P5 H14	Upland	NaCRRI	UG
14	NERICA 6	Lowland	ARC	ARC	44	P27 H3	Upland	NaCRRI	UG
15	1189 LINE	Lowland	ARC	ARC	45	ART3 -7L3P3-B-B-2	Upland	ARC	ARC
16	1191 LINE	Lowland	ARC	ARC	46	P23 H1	Upland	NaCRRI	UG
17	326104 LINE	Lowland	ARC	KR	47	ART3-8L6P3-2-3-B	Upland	ARC	ARC
18	Supa TZ	Lowland	Tanzania	TZ	48	Mbume	Upland	Landrace	LDR
19	Basmati 370	Lowland	IRRI	IRRI	49	ART25-3-29-2-B	Upland	ARC	ARC
20	SK-95-4	Lowland	Mali	IER	50	ART3-8L6P3-2-2-B	Upland	NaCRRI	UG
21	SK-7-8	Lowland	Mali	IER	51	ART12-L2P2-20-3-1-1	Upland	ARC	ARC
22	BR4	Lowland	Landrace	LDR	52	P24 H1	Upland	ARC	ARC
23	BG 400–1	Lowland	Landrace	LDR	53	P62 H17	Upland	NaCRRI	UG
24	NAMCHE 6	Upland	NaCRRI	UG	54	ART16-4-11-13-4	Upland	NaCRRI	UG
25	NAMCHE 1	Upland	NaCRRI	UG	55	PCT-4\0\0\0˃19-M-1-1-5-1-M	Upland	ARC	ARC
26	NAMCHE 3	Upland	NaCRRI	UG	56	NERICA 4	Upland	ARC	ARC
27	NAMCHE 2	Upland	NaCRRI	UG	57	DKAP-27	Upland	Mali	IER
28	Namche 4	Upland	NaCRRI	UG	58	NERICA 1	Upland	ARC	ARC
29	Namche 5	Upland	NaCRRI	UG	59	NERICA 10	Upland	ARC	ARC
30	SCRIDO 37-4-2-2-5	Upland	Madagascar	MDG					

^Ɨ^ PID = Population Identity; IRRI, International Rice Research Institute; ARC, Africa Rice Centre; UG, National Crops Resources Research Institute(NaCRRI)-Uganda; IER, Institut d’Economie Rurale –Mali, CIAT, International Center for Tropical Agriculture; MDG, Madagascar;TZ, Tanzania;LDR, Landrace-South Sudan;

### Quantification of amylose and amylopectin

Amylose and amylopectin content of the starch was determined by the method of Gibson et al. [37] using a Megazyme amylose/amylopectin assay kit (K-AMYL 04/06, Megazyme International Ireland Ltd., Co. Wicklow, Ireland), which is a modification of a Con A method developed by Yun and Matheson [18]. The method is also modified from Morrison and Laignelet [38] and uses an ethanol pre-treatment step to remove lipids prior to analysis. Initially, rice samples were dehusked and polished prior to milling. Twenty whole-milled rice kernels from each of the 36 rice genotypes were ground separately and accurately weighed (20–25 mg to the nearest 0.1 mg) into a 10 ml screw capped Kimax sample tube. One millilitre of dimethyl sulfoxide (DMSO) was added while gently stirring at low speed on a vortex mixer. Samples were heated in a boiling water bath for 15 minutes with intermittent high-speed stirring on a vortex mixer and allowed to cool for 5 minutes at room temperature. Two millilitres of 95% ethanol were added with continuous stirring on a vortex mixer. A further 4 millilitres of ethanol were added and allowed to mix and kept overnight or allowed to stand for 15 minutes. After precipitate formation, the tubes were centrifuged for 5 minutes at 2000 revolutions per minute (rpm), and supernatant discarded. Two millilitres DMSO was then added to the pellet with vortexing and heating in boiling water bath for another 15 minutes. Four millilitres of Con A solvent was immediately added and solution adjusted to 25 ml in volumetric flask by repeated washing with Con A solvent (this was labelled solution A). One millilitre of solution A was then pipetted into a 2 ml eppendorf microfuge tube with the addition of 0.5 ml Con A solution and allowed to stand at room temperature for one hour. The Eppendorf tubes were then centrifuged for 10 minutes at 14000 rpm at room temperature. One millilitre of supernatant was transferred to a 15 ml centrifuge tube and 3 ml of sodium acetate buffer of pH 4.5 added. The tubes were heated in a boiling water bath for 5 minutes and allowed to equilibrate in a 40°C water bath for 5 minutes. About 0.1 ml of amyloglucosidase/α-amylase enzyme mixture was added and incubated at 40°C for 30 minutes. The tubes were then centrifuged at 2000 rpm for 5 minutes. To 1.0 ml aliquots of the supernatant, 4 ml of GOPOD reagent was added and incubated at 40°C for 20 minutes. The absorbance of each sample and the D-glucose controls were read at 510 nm against the reagent blank. Total starch absorbance was determined by mixing 0.5 ml aliquots of solution A with 4 ml of sodium acetate buffer. A 0.1 ml of amyloglucosidase/ α -amylose solution was added and incubated for 10 minutes at 40°C. One millilitre aliquots of this solution was transferred to glass test tubes, to which 4 ml GOPOD reagent was added and incubated for 20 minutes at 40°C. The incubation was performed concurrently with the samples and standards. Absorbance of samples was read at 510 nm. Amylose content was then determined as follows;

$$\begin{array}{ll} \text{Amylose},\;\%\;\left(\text{w}/\text{w}\right) & =\frac{\text{Absorbance}\;\left(\text{Con}\;\text{A}\;\text{Supernatant}\right)x\;6.15\;x\;100}{\text{Absorbance}\;\left(\text{Total}\;\text{Starch}\;\text{Aliquot}\right)9.2\;\text{x}\;1}\\ & =\frac{\text{Absorbance}\;\left(\text{Con}\;\text{A}\;\text{Supernatant}\right)x\;66.8}{\text{Absorbance}\;\left(\text{Total}\;\text{Starch}\;\text{Aliquot}\right)} \end{array}$$

where, 6.15 and 9.2 are dilution factors for the Con A and Total Starch extracts, respectively. The samples were then classified following standard procedures by Juliano [39] with slight modifications, where; 3–9% amylose content indicates waxy to very low AC, 10–19% amylose content indicates low AC; 20–25% amylose content indicates intermediate AC, 26–30% amylose content indicates high-AC, while >31% amylose content indicates very high-AC.

### Measurement of gelatinization temperature

Gelatinization temperature (GT) was assessed indirectly as the alkali spreading value of hulled kernels as per modified procedure of Little et al. [40]. Twelve whole grains, were immersed in petri-plates containing 1.7% KOH in such a way that no two grains were in contact with each other. The plates were then incubated for 24 h at room temperature. The ASV were determined by visual scoring of the appearance of the grains and disintegration on a 1–7 linear scale as described by Govindaraj et al. [41], where; 1 = grains not affected, 2 = grains swollen, 3 = grains swollen, collar incomplete and narrow, 4 = grain swollen, collar complete and wide, 5 = grains split or segmented, collar complete and wide, 6 = grain dispersed, merging with collar and 7 = grain completely dispersed and intermingled. Grains swollen to the extent of a cottony centre and a cloudy collar were given an ASV score 4 and used as a check for scoring the rest of the samples. Since ASV is inversely related to GT the higher value of ASV was taken for low GT and vice versa. A rating of 1.00–2.99 was taken as high GT (>74°C), 3.00–4.99 as intermediate (69–74°C) and 5.00–7.00 as low GT (55–68°C) as referred in Govindaraj et al. [41].

### DNA isolation and genotyping

Total genomic DNA was isolated from leaves of three-week old plants using the ZYMO research *Quick*-DNA^™^ Plant/Seed 96 Kit, where a single individual plant was considered for each genotype. Subsequently, 40 μl of a 50 ng/μl DNA of each sample were sent to Diversity Arrays Technology (DArT) Pty Ltd, Australia (http://www.diversityarrays.com/dart-map-sequences) for whole genome scan using Diversity Arrays Technology (DArT) markers. Whole-genome genotyping for the 59 rice genotypes was carried out using Genotyping-By-Sequencing (GBS) technology as described by Elshire et al. [24] using 18,927 DArT markers. The markers were integrated into a linkage map by inferring marker order and position from the consensus DArT map.

### Data filtering process and DArTseq SNP calling

**Fig 1:**
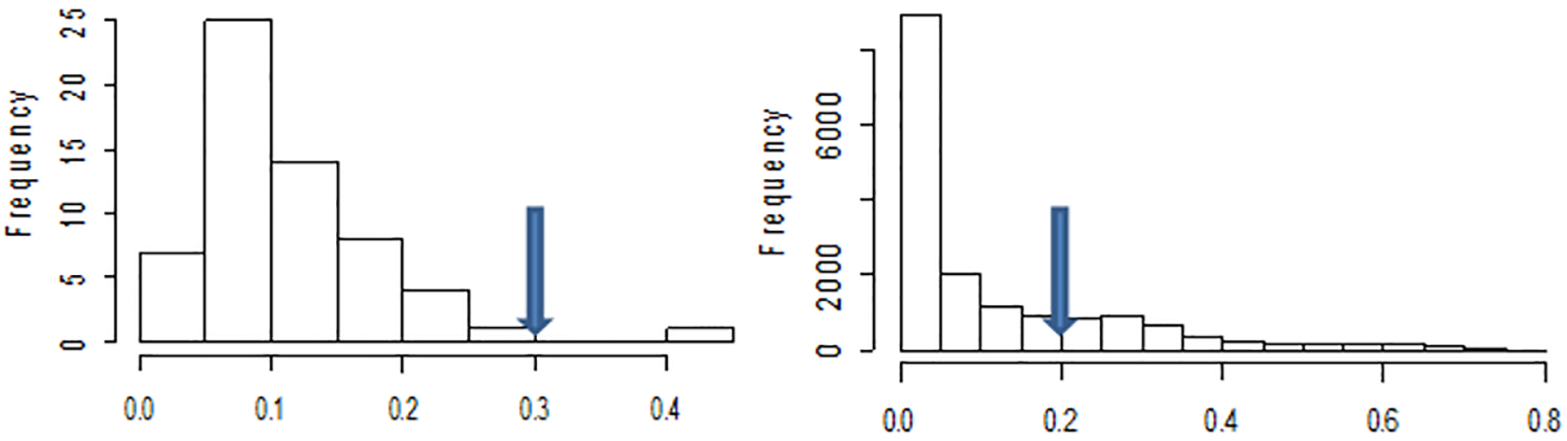
Frequency of genotypes with missing data (left), and frequency of DArTseq SNPs (loci) with missing data (right).

DArTseq SNP derived markers were filtered to remove bad SNPs and genotypes using PLINK 1.9 software in MS window and R statistical software, where genotypes with > 30% missing data, SNP loci with >20% missing data (Fig 1) and rare SNPs with <5% minor allele frequencies (MAF) were pruned. Only 525 DArTseq informative SNPs and 59 genotypes were considered after filtering and data quality control process.

## Statistical analyses

### Population structure analysis

We investigated the genetic structure and relationship among 59 rice genotypes using 525 DArTseq-derived SNP markers distributed across the rice genome as described by Pritchard et al. [42]. Bayesian clustering method was applied to identify clusters of genetically similar individuals using the software STRUCTURE version 2.3 [43]. Cluster values (K) ranging from 1 to 10, and ten independent runs were used for each value in order to obtain consistent results. The best K-value for estimating a suitable population size for the dataset was determined as K = 2 based on the Evanno et al. [44] method from STRUCTURE run. In addition, population differentiation due to genetic structure was assessed using a Neighbour Joining (NJ) tree method [45] and Principal Component Analysis (PCA) generated by R statistical software. Analysis of molecular variance (AMOVA) and genetic diversity was performed using GenAlEx V6.5 software [46]. DArTseq SNP data were numerically coded as follows: A = 1, C = 2, T = 3, G = 4 and missing data was coded as 0 (S1 File) as suggested in GenAlEx V6.5 user manual.

### Linkage disequilibrium

Linkage disequilibrium analysis was performed using TASSEL V5.3.1 software [47] with selected 525 DArTseq-derived SNP markers of known position [48] out of the complete set of 18,927 polymorphic markers. Linkage disequilibrium was estimated as squared allele frequency correlations (*R*^*2*^), and only *P*-values ≤0.01 for each pair of loci were considered significant.

### Association mapping

Determinants of grain quality including grain length (GL), grain width (GW), grain length/width ratio (L/W), amylose content (AC) and gelatinization temperature (GT) were considered for association mapping. Association mapping analysis was performed with TASSEL V5.3.1 software [47] using both the General Linear Model (GLM) and Mixed Linear Model (MLM) methods. Two different methods were considered for both GLM and MLM; where, for GLM, the model with no control for population structure and relatedness (naive model), and the model with population structure (the Q model) were performed, whereas for MLM; the model that considers the familial relatedness between accessions (the K model), and the model that takes into account both the population structure and the familial relatedness were used, that is, Q + K model as described by Yu et al. [49]. Where, the general equations for GLM and MLM are: y = Xa + e; and y = Xa+ Qb+ Zu + e, respectively; where, y is vector for phenotypes; a is the vector of marker fixed effects, b is a vector of fixed effects, u is the vector of random effects, and e is the vector of residuals. X denotes the genotypes at the marker; Q is the Q-matrix obtained from the STRUCTURE software and Z is an identity matrix. Both models were applied with and without considering the fixed effect of the population structure. Marker alleles with P-values ≤0.001 in both MLM and MLM-Q models were declared significantly associated with grain quality parameters.

## Results

### Genetic diversity analysis based on geographic origin

The number of accessions, number of alleles, genetic diversity, heterozygosity, polymorphism information content (PIC) and major allele frequency of the eight populations is shown in Table 2. The mean PIC values for each SNP locus in rice collections from ARC, CIAT, IER, IRRI, LDR, MDG, TZ and UG were 0.34, 0.02, 0.27, 0.29, 0.23, 0.10, 0.06 and 0.34, respectively. The mean number of alleles for each population was 2.0, 1.05, 1.94, 1.90, 1.77, 1.28, 1.19 and 2.0 respectively. The tendencies of PIC and mean number of alleles were in the order ARC = UG > IRRI > IER > LDR > MDG > TZ > CIAT, respectively. Rice population from ARC had the highest level of PIC, gene diversity and mean number of allele, but lowest level of major allele frequency (0.64). Rice population from CIAT had the lowest level of PIC, gene diversity and mean number of allele, but the highest level of major allele frequency (0.98).

**Table 2:** Estimation of gene diversity, heterozygosity, PIC and major allele frequency in 59 rice accessions.

Group	No. of accessions	Allele.No	Gene Diversity	Heterozygosity	ƗPIC	Major Allele Frequency
**ARC**	22.00	2.00	0.45	0.09	0.34	0.64
**CIAT**	1.00	1.05	0.02	0.05	0.02	0.98
**IER**	4.00	1.94	0.34	0.08	0.27	0.76
**IRRI**	4.00	1.90	0.37	0.07	0.29	0.71
**LDR**	3.00	1.77	0.29	0.20	0.23	0.78
**MDG**	2.00	1.28	0.14	0.06	0.10	0.87
**TZ**	2.00	1.19	0.08	0.09	0.06	0.94
**UG**	21.00	2.00	0.44	0.14	0.34	0.66

ARC, Africa Rice Centre; CIAT, International Center for Tropical Agriculture; IER, Institut d’Economie Rurale –Mali; IRRI, International Rice Research Institute; LDR, Landrace-South Sudan; MDG, Madagascar; TZ, Tanzania; UG, National Crops Resources Research Institute-Uganda (NaCRRI);

^Ɨ^ Polymorphism information content

### Population structure and genetic relationships

**Fig 2:**
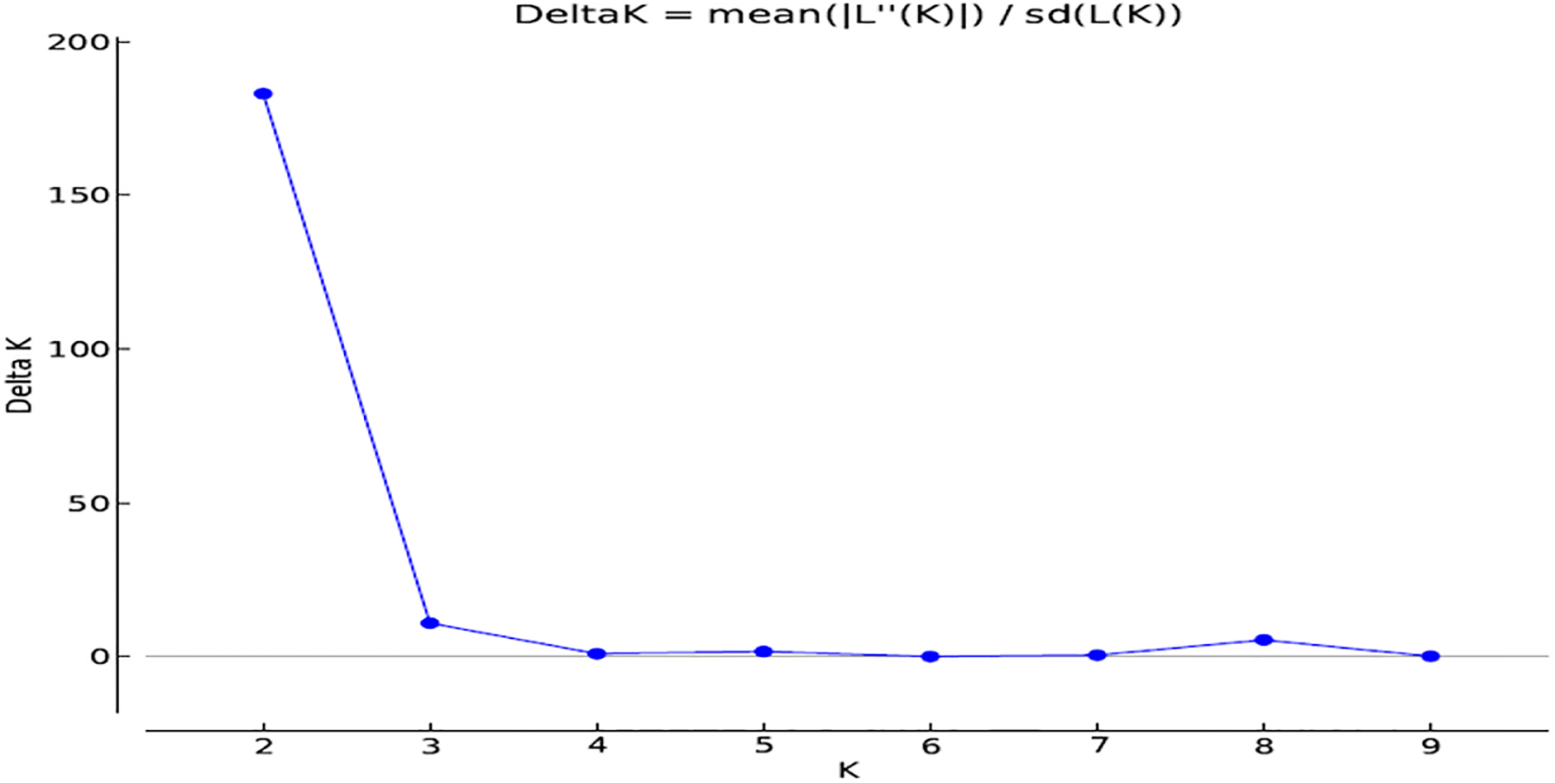
Magnitude of Δ K as a function of Delta K for 59 rice genotypes based on 525 polymorphic DArTseq-derived SNP markers.

**Fig 3:**
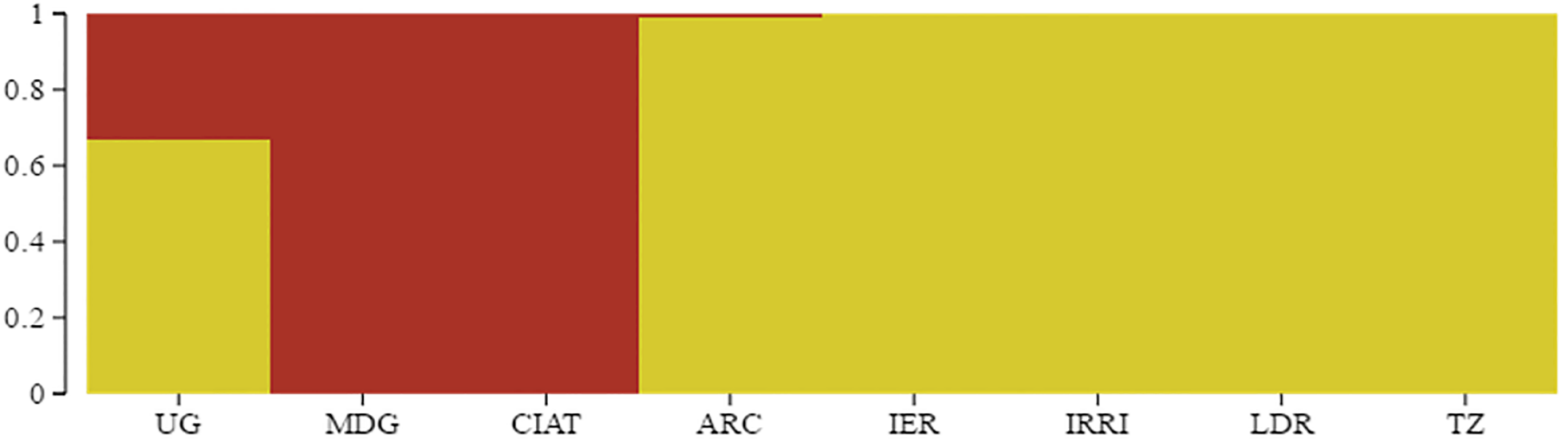
Distribution pattern of 59 rice genotypes based on Bayesian clustering method of DArTseq derived-SNP markers.

**Fig 4:**
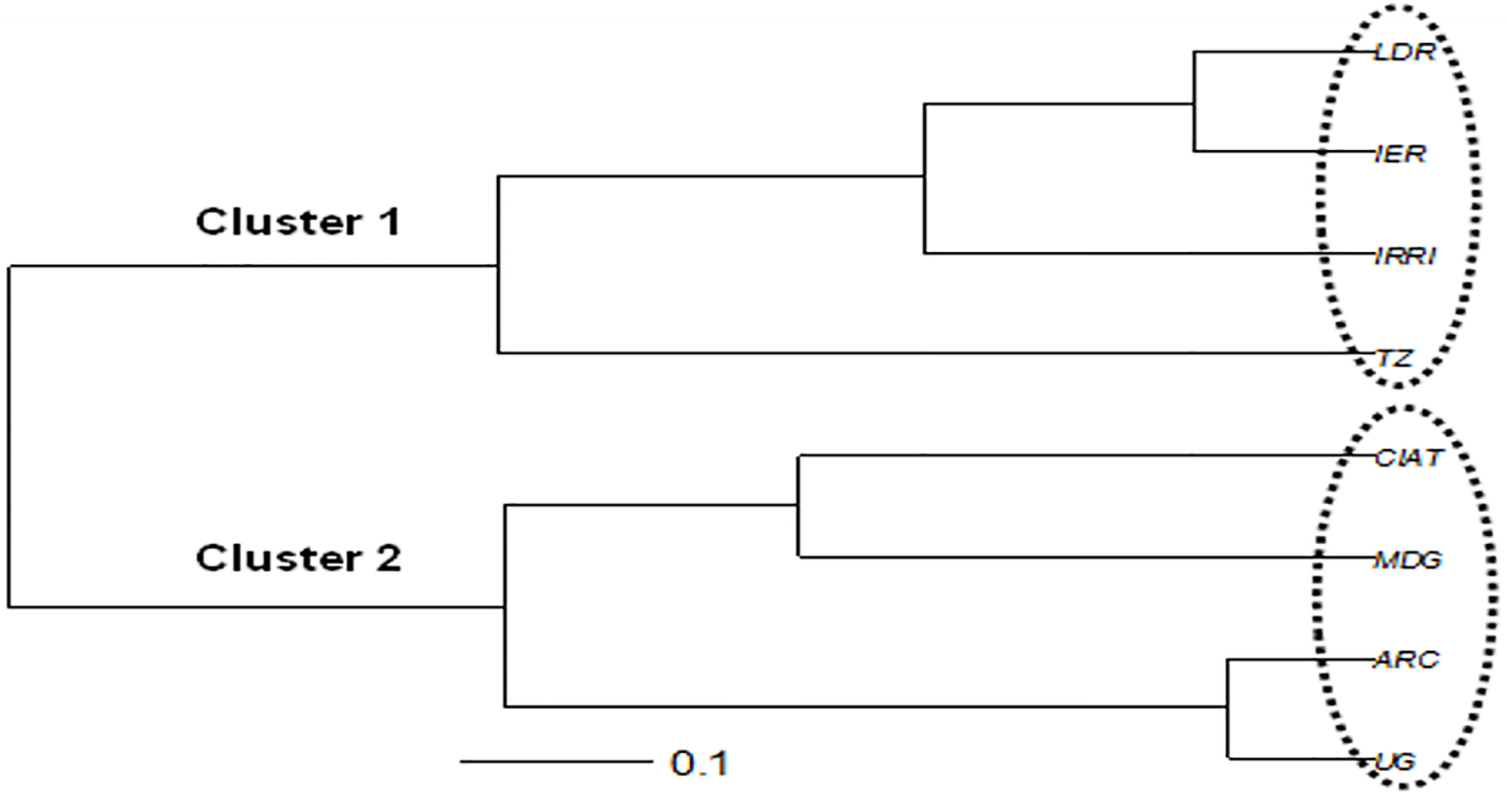
Dendrogram of a Neighbor-Joining (NJ) tree of rice populations constructed for 59 rice genotypes using DArTseq markers based on a mean fixation index (F_st_) estimate value of 0.134.

Results of population structure analysis of 59 rice genotypes using a model-based program, STRUCTURE, for *K* ranging from 1 to 10, and by inferring on Delta K of Evanno et al. [44] identified the most suitable K value for determining the genetic cluster as K = 2 (Fig 2). The number of populations were visualized using Structure Plot V2.0 [50], where genotypes that scored >0.80 were considered as pure and <0.80 as admixture (Fig 3). Only genotypes with origin from the National Crops Resources Research Institute-Uganda (UG) suggested considerable degree of admixtures (<80%). Two major clusters were formed where genotypes from UG, Africa Rice Centre (ARC), Madagascar (MDG) and International Centre for Tropical Agriculture (CIAT) formed the first cluster, while genotypes from International Rice Research Institute (IRRI), Tanzania (TZ), Institut d’Economie Rurale-Mali (IER) and landraces from South Sudan (LDR) comprised the second cluster. Similarly, using Neighbour Joining (NJ) method and based on a mean fixation index (F_st_) estimate value of 0.134 generated by PLINK 1.9 software, genotypes were grouped into two major clusters (Fig 4), confirming the results of population structure analysis. Cluster 1 assembled genotypes from UG, ARC, MDG and CIAT, while cluster 2 grouped together genotypes from IRRI, TZ, IER and LDR.

### Principal component analysis

**Fig 5:**
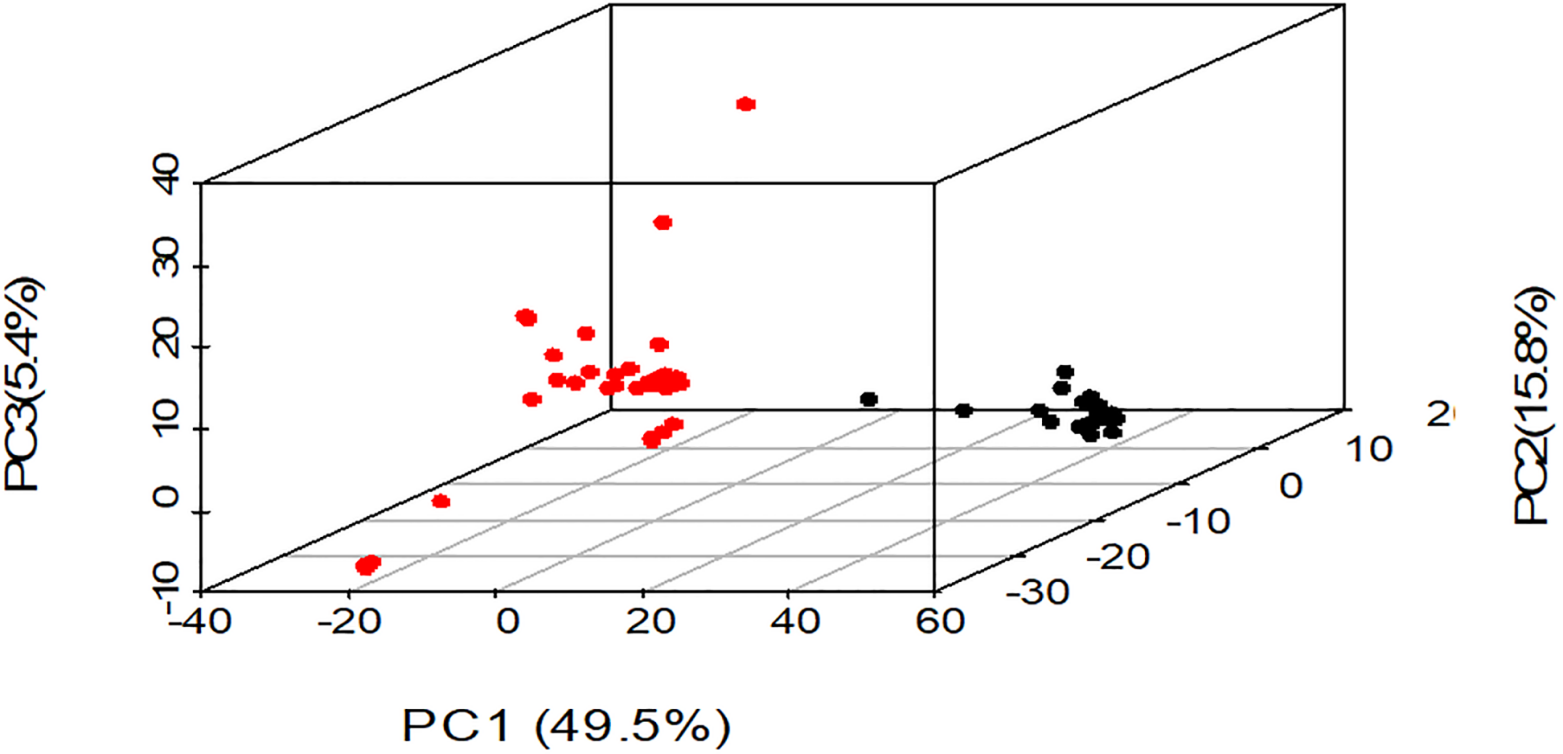
3D scatter plot of principal component analysis for 59 rice genotypes based on DArTseq-derived SNP markers.

Using a 3D scatter plot of principal component analysis (PCA) and based on 525 DArTseq SNPs, two major clusters were clearly distinguished among all rice populations (Fig 5) consistent with results from population structure analysis. Rice genotypes from cluster 1 were depicted by red colour, while cluster 2 genotypes were represented by black colour. Principal component analysis yielded three principal components accounting for 70.7% of total variance observed. Breakdown of this cumulative variance value revealed contributions of 49.5%, 15.8% and 5.4% for PCA1, PCA2 and PCA3, respectively.

### Genetic distance among populations

The genetic distance among the different populations was estimated with 525 DArTseq-derived SNP markers (Table 3). The greatest genetic distance was observed between genotypes from TZ and CIAT populations (0.865) and between genotypes from TZ and MDG populations (0.808). In addition, the least genetic distance was observed between genotypes from LDR and IER populations (0.004) and between genotypes from LDR and IRRI populations (0.017).

**Table 3:** Genetic distances between different populations.

Populations	ARC	CIAT	IER	IRRI	LDR	MDG	TZ
CIAT	0.067	0	-	-	-	-	-
IER	0.213	0.481	0	-	-	-	-
IRRI	0.157	0.370	0.028	0	-	-	-
LDR	0.237	0.526	**0.004**	**0.017**	0	-	-
MDG	0.157	0.686	0.494	0.463	0.582	0	-
TZ	0.377	**0.865**	0.082	0.112	0.049	**0.808**	0
UG	0.023	0.228	0.218	0.194	0.267	0.069	0.399

ARC, Africa Rice Centre; CIAT, International Center for Tropical Agriculture; IER, Institut d’Economie Rurale –Mali; IRRI, International Rice Research Institute; LDR, Landrace-South Sudan; MDG, Madagascar; TZ, Tanzania; UG, National Crops Resources Research Institute-Uganda (NaCRRI);

### Analysis of molecular variance

Analysis of molecular variance (AMOVA) among the 59 rice genotypes indicated that 11.24% of the variance was due to genetic differentiation among the populations, 67.30% of the variance was accounted by genetic differentiation among individuals within populations, while the remaining 21.46% of the variance was due to the differences within individuals (Table 4).

**Table 4:** AMOVA of a panel of 59 rice genotypes.

Source of variation	d.f	Sum of squares	Variance components	Percentage variation
Among populations	7	2690.60	14.80	11.24
Among individuals within populations	51	10485.42	88.66	67.30
Within individuals	59	1668.00	28.27	21.46
Total	117	14844.02	131.74	

### Phenotypic distribution of grain quality traits

**Fig 6:**
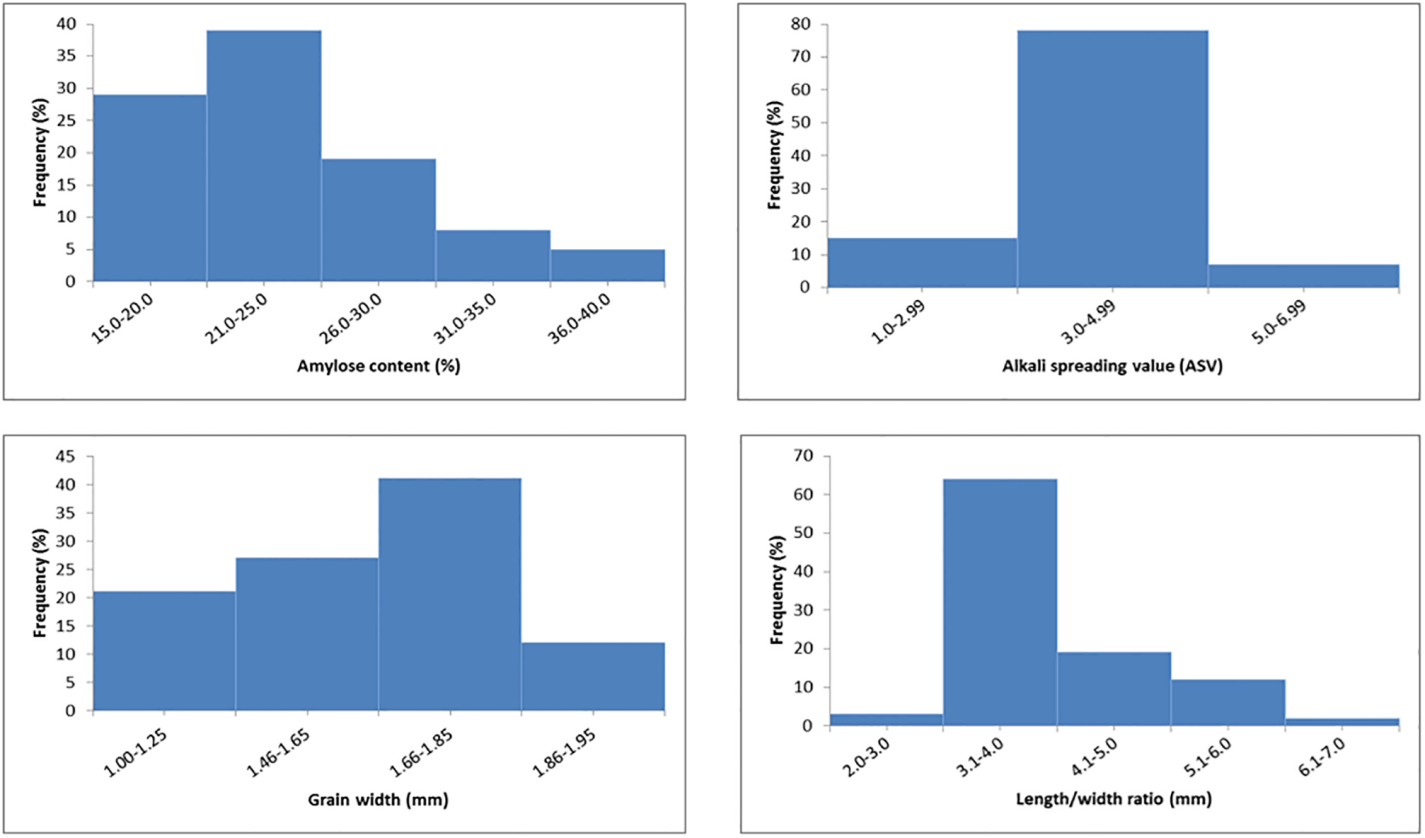
Phenotypic distribution of GWAS results for grain quality traits (AC, amylose content; ASV, alkali spreading value; GW, grain width; L/W, grain length to width ratio); grain shape (length/width ratio): slender≥3.0; Medium = 2.1–3.0; Bold = 1.1–2.0; Round<1.1; Grain length: Extra-long(≥7.5 mm); Long (6.6–7.5 mm); Medium (5.51–6.6 mm); Short (<5.51mm).

Grain shape (measured as the grain length-to-width ratio) and starch related qualities such as amylose content and gelatinization temperature (measured indirectly as alkali spreading value (ASV)), are the main properties considered for selecting breeding lines with improved quality [51]. In this study phenotypic distribution for the aforementioned grain quality traits were determined among 59 rice genotypes. Analysis of the frequency distributions of the phenotypic classes suggested that all traits were quantitative and continuous (Fig 6). Frequency distributions suggested an overall broad variability, which is ideal to be efficiently exploited in GWAS studies. All phenotypic traits were approximately normally distributed (Fig 6); a few distributions, though, were found to be slightly skewed (amylose content, grain width and length to width ratio), but none showed a clear separation in two or more classes. Grain length varied from 5.0–7.95 mm, where most of the genotypes were characterized as long grains. Grain shape ranged from 2.0–7.0 and majority of the genotypes were categorized as slender grains. The ASV varied from 1.0–6.99 which relates to high-low gelatinization temperature (GT) and most of the genotypes were grouped as intermediate GT. Percent AC ranged from 15 to 40% where majority of the genotypes were classified as intermediate AC.

### Genome-wide association scans for grain quality traits

**Fig 7:**
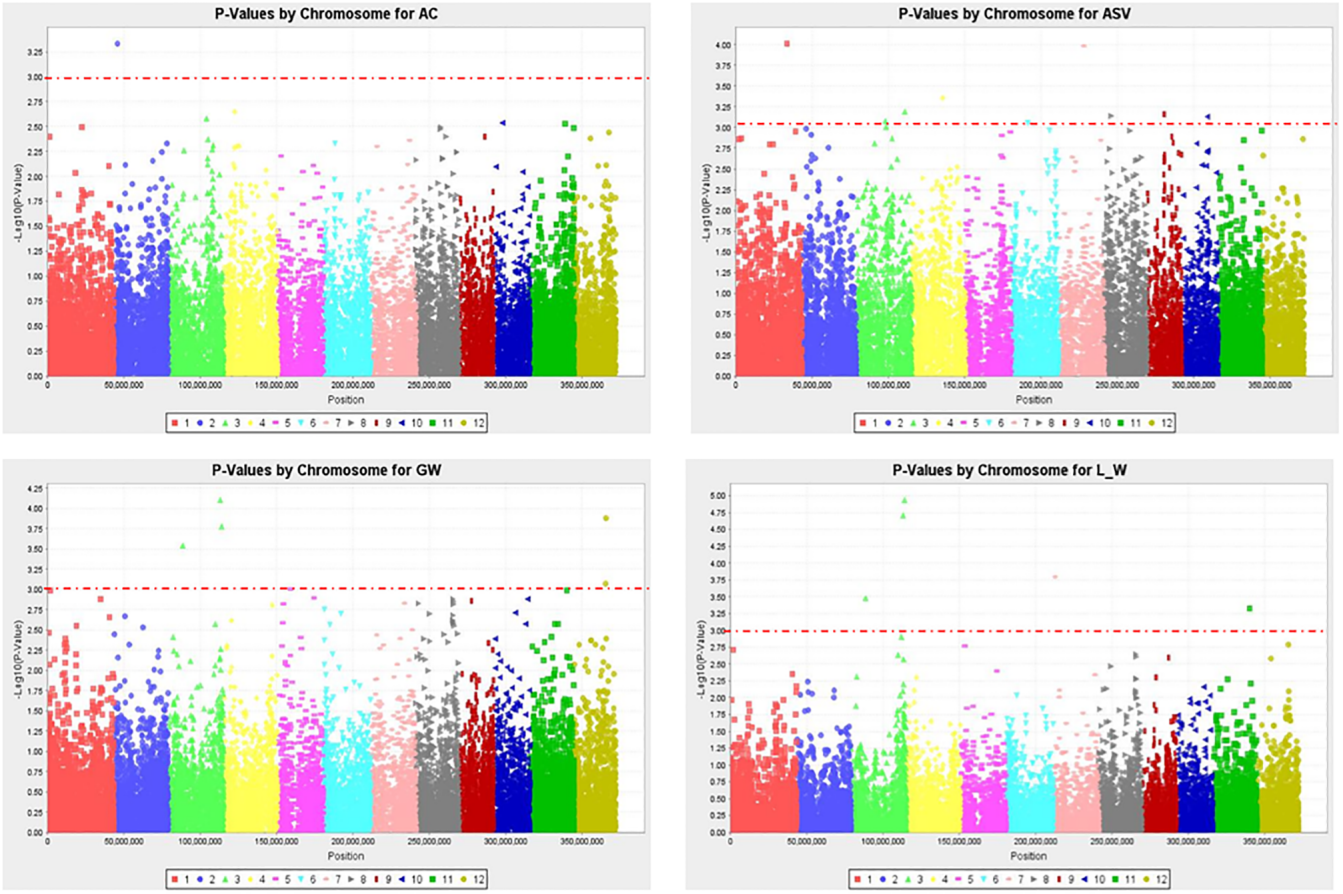
Manhattan plots of GWAS results for grain quality traits (AC, amylose content; ASV, alkali spreading value; GW, grain width; L_W, grain length to width ratio); Threshold = −log_10_(p−value) > 3.

**Fig 8:**
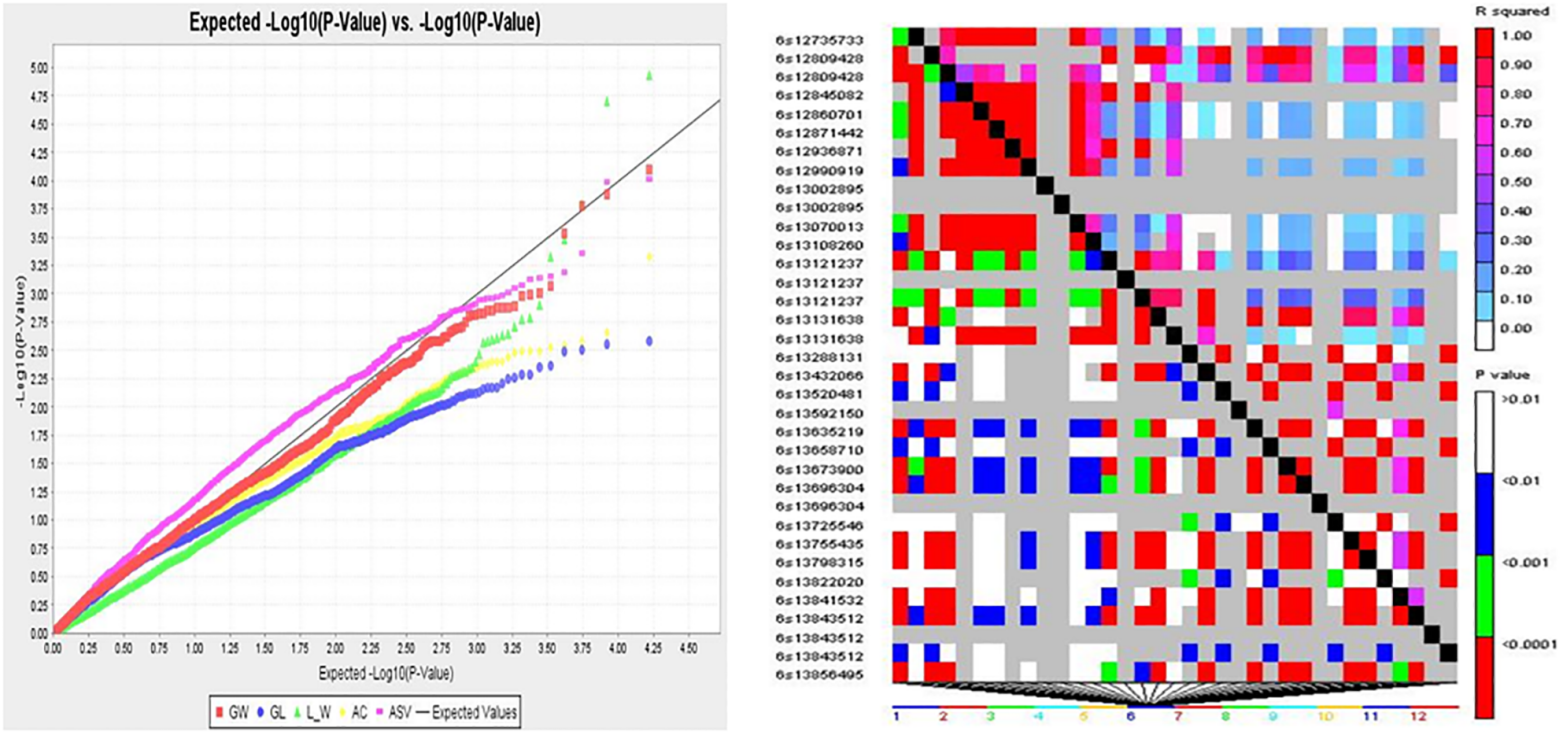
Q-Q plot (left) and patterns of LD blocks (right) of GWAS results indicating the position of candidate genes and/or QTL regions associated with grain quality traits.

Determinants of grain quality including grain length (GL), grain width (GW), grain length/width ratio (L/W), amylose content (AC) and gelatinization temperature (GT) were considered for genome-wide association studies (GWAS) using 525 DArTseq SNP derived markers. Association mapping analysis was performed with TASSEL V5.3.1 software [47] using both the General Linear Model (GLM) and Mixed Linear Model (MLM) methods. Both known associations (for GW, L/W, AC and ASV) as well as candidate loci were identified, where *P*-values were used to determine the association of QTLs with markers while percent variance explained (PVE) predicted the magnitude of QTL effects. Manhattan plots for grain quality traits were generated in GWAS indicating the most significant associations ((−log (*p*-value)>3)) (Fig 7). A quantile-quantile (Q-Q) plot confirmed a normal distribution of phenotypic traits while the pattern of linkage disequilibrium (LD) blocks suggested the extent of association mapping, where the red sites represented SNPs that are in high linkage disequilibrium with each other and thus inherited together (Fig 8). A total of 22 significant (*P* < 0.001) association signals were detected for grain quality traits (Table 5). For AC, one QTL was identified on chromosome 2 that explained 48% of phenotypic variation. Our study did not detect any significant AC QTLs at the interval corresponding to the Wx gene on chromosome 6. Ten QTLs were identified for ASV on chromosomes 1, 3, 4, 6, 7, 8, 9 and 10, contributing 19–31% of phenotypic variance. Six QTLs were also detected for GW on chromosomes 3, 5 and 12, which individually explained 23–43% of phenotypic variance. Furthermore, five QTLs were identified for L/W on chromosomes 3, 7 and 11 contributing 20–35% of phenotypic variance. SNP12_100006178, SNP13_3052560 and SNP14_3057360 (highlighted in bold) individually co-localised with two functional gene groups that are associated with QTLs for GW and L/W on chromosome 3 (Table 5). The AC allele (C/T) was traced back to parent K5; ASV alleles (G/A, A/G) were located in parents ART2-4L3P1-2-1, BG400-1, JARIBU and SUPA TZ; while the co-localised QTLs for GW and L/W came from JARIBU, BR4 and ART3-8L6P3-2-2-B. In general, 2 of the 22 associations identified were in regions where the QTL associated with the given traits had been reported in previous studies (http://www.gramene.org/ (Table 6)); the other 20 significant SNP loci are potential novel QTLs.

**Table 5:** Genome wide significant associations (R^2^) of single nucleotide polymorphisms (SNPs) with amylose content (AC), alkali spreading value (ASV), grain width (GW) and grain length to width ratio (L/W).

S/No.	Grain quality trait	Marker	Chromosome	Position (cM)	Ɨ Allele	*p*-value	PVE
1	AC	SNP1_3444982	2	16.10	C/T	4.68E-04	0.48
2	ASV	SNP2_5143094	1	144.73	G/A	9.77E-05	0.27
3	ASV	SNP3_3438578	3	163.67	A/G	6.45E-04	0.24
4	ASV	SNP4_5142766	3	106.19	G/A	8.34E-04	0.31
5	ASV	SNP5_3453263	3	106.29	A/G	9.77E-04	0.26
6	ASV	SNP6_3053581	4	45.43	A/G	4.37E-04	0.27
7	ASV	SNP7_3755646	6	55.72	C/T	8.81E-04	0.19
8	ASV	SNP8_9752845	7	80.62	G/A	1.03E-04	0.27
9	ASV	SNP9_3049990	8	34.13	G/A	7.19E-04	0.21
10	ASV	SNP10_13890040	9	25.24	C/G	6.93E-04	0.25
11	ASV	SNP11_3053914	10	49.59	G/A	7.33E-04	0.24
12	**GW**	**SNP12_100006178**	**3**	**179.45**	**C/T**	**7.93E-05**	**0.32**
13	**GW**	**SNP13_3052560**	**3**	**184.08**	**A/C**	**1.67E-04**	**0.28**
14	**GW**	**SNP14_3057360**	**3**	**55.35**	**C/T**	**2.90E-04**	**0.23**
15	GW	SNP15_3049175	5	52.50	T/A	9.82E-04	0.24
16	GW	SNP16_100003971	12	64.57	C/T	1.33E-04	0.43
17	GW	SNP17_3448915	12	63.67	A/C	8.55E-04	0.37
18	**L/W**	**SNP13_3052560**	**3**	**184.08**	**A/C**	**1.17E-05**	**0.32**
19	**L/W**	**SNP12_100006178**	**3**	**179.45**	**C/T**	**1.99E-05**	**0.35**
20	**L/W**	**SNP14_3057360**	**3**	**55.35**	**C/T**	**3.33E-04**	**0.20**
21	L/W	SNP18_100004705	7	9.27	C/T	1.60E-04	0.23
22	L/W	SNP19_9755868	11	80.06	C/A	4.67E-04	0.20

^Ɨ^ Allele corresponding to grain quality trait based on 59 rice genotypes;

PVE, percent variance explained

**Table 6:** Two of the 22 associations previously reported for grain quality traits.

S/N0.	species	Trait name	Trait synonyms	Linkage group	Trait symbol	Published symbol	Qtl accession id	Start position (cM)	Stop position (cM)	Reference
1	*Oryza sativa*	grain width	KW, kernel width, width of cooked rice, width of milled rice	5	GRWD	-	CQAL27	30.2	66	[52]
2	*Oryza sativa*	length to width ratio	LWR, grain shape, length:width ratio of the rice grain	3	GRLGWDRO	lwr3.1	AQFA014	31.2	76.9	[21]

Source: http://www.gramene.org/

## Discussion

Genome level profiling of rice germplasm collections is a critical initial step in identification of divergent parents for effective utilization in rice breeding programs. The present study is the first major effort to perform genetic diversity studies and population structure analysis on a panel of 59 rice germplasm collections in South Sudan for effective breeding.

Our study highlights the potential of highly informative and selective DArTseq-derived SNP markers for genetic diversity analysis and genome wide association studies in rice. Results of the diversity analysis based on geographical origin (Table 2) indicated that rice collections of ARC population had the highest polymorphic information content and number of alleles similar to UG population. The values were intermediate for IRRI, IER, LDR, MDG and low for TZ and CIAT populations (Table 2). These results suggested that most of the rice genotypes in South Sudan are largely adopted from West Africa where the Africa Rice Centre (ARC) gene bank is entrusted with collection, conservation and utilization of most African rice genetic resources [53]. Hence, a large number of the rice germplasm from ARC have spread to other countries within Africa such as Uganda (UG), Mali (IER), Madagascar and South Sudan. A few of the rice genotypes including accessions from IRRI and CIAT originated mainly from Asia and Latin America respectively as depicted by their geographical location.

Results of population structure analysis (Figs 2, 3 and 4) revealed only two major clusters and indicated a clear genetic divergence based on origin and breeding history of the rice genotypes, confirming results from principal component analysis. Genotypes were grouped into two distinct clusters based on environmental adaptation, pedigree information and genetic distances. A low mean fixation index (F_st_) estimate value of 0.134 and a small percentage variation (11.2%) among populations as revealed by analysis of molecular variance (Table 4) suggested a low degree of differentiation among populations and increased levels of admixtures. Low F_st_ estimate values ranging between 0.047–0.192 were reported by Oloka et al. [54] for rice populations sampled from IRRI, AfricaRice and NaCRRI-Uganda, and by Ogunbayo et al. [55] for genotypes originating from AfricaRice. Semon et al. [56] and Wang et al. [57] suggested that the domestication of African rice may have been influenced by the introduction of Asian rice into West Africa and subsequent intercrossing. In particular, the rice population from Uganda indicated a high level of admixtures due to the on-going breeding activities. Oloka et al. [54] reported similar findings on rice diversity studies in Uganda. Thus based on the genetic distances between different populations, genotypes were clustered according to genetic relatedness where one cluster comprised accessions from CIAT, ARC, MDG and UG, while the other consisted of genotypes from IRRI, IER, LDR and TZ.

Analysis of frequency distributions of phenotypic classes (Fig 6) indicated that all the grain quality traits in this study were quantitative and continuous which is in agreement with other previous studies [58, 59, 60]. In addition, most of the genotypes were categorized as long and slender grains, with intermediate gelatinization temperature and amylose content. Consequently, based on the desirable characteristics of the aforementioned genotypes, they may be considered potential high market value rice grains with improved eating and cooking properties [61].

We identified twenty-two significant associations with PVE of between 19–48% for rice grain quality traits in the entire set of genotypes, including 1 association with AC, 10 associations with ASV, 6 associations with GW and 5 associations with L/W (Table 5). In the present study, no significant SNP associations were detected in the interval corresponding to the Wx gene on chromosome 6. This was probably due to low DArT SNP marker density and uneven distribution of the DArT SNP markers or the difference in AC gene for different genotypes. In addition, previous reports on QTL analysis for rice grain quality traits [52, 59] suggested the complex nature of grain quality traits and that several chromosomal regions were involved in the expression of a phenotype. Several of the significant SNP loci were located on chromosome 3, which had previously been identified as a rice grain shape QTL hotspot region [62]. Two of the 22 significant associations were in chromosomal regions in which rice grain shape QTLs had previously been located (http://www.gramene.org/). The other 20 significant SNP loci suggested the likelihood discovery of novel alleles associated with rice grain quality traits. Furthermore, SNP12_100006178, SNP13_3052560 and SNP14_3057360 individually co-localised with two functional gene groups that were associated with QTLs for grain width and grain length to width ratio on chromosome 3, indicating trait dependency or pleiotropic-effect loci. Hu et al. [62] identified six chromosomal regions on chromosomes 1, 2, 3, 5 and 6 that had pleiotropic effects on two or more determinants of rice grain shape. Biscarini et al. [63] also identified several significant associations that co-localised with QTLs and candidate genes influencing the phenotypic variation of single or multiple rice grain quality traits. These findings provide a direction to effectively exploit genetic hot-spot regions overlapping for multiple traits to enhance predictability of superior lines in a rice breeding population. Furthermore, our results might increase the descriptive power of QTLs associated with grain quality traits in rice and thus provide useful information for further fine mapping and cloning.

## Conclusion

The present study demonstrates the potential of highly informative and selective DArTseq-derived SNP markers for genetic diversity analysis and genome wide association studies in the tested rice genotypes. This study also provides a direction for breeding efforts in the selection of parents from the current collection with potential for novel genes or QTLs for important agronomic traits. A low degree of differentiation among sampled populations suggested the need for widening on the genetic base through the introduction of distant or wild relatives. However, the study also indicated that wide variability exists in the current rice germplasm collections for grain quality traits probably due to intercrossing between populations. Genome-wide association studies successfully identified and tagged 22 DArTseq-derived SNP loci significantly associated with rice grain quality traits. Among these, two SNP loci were found in regions where the QTL associated with the given traits had previously been reported, while the other 20 significant associations were indicative of the likelihood discovery of novel alleles associated with rice grain quality traits. Significant QTL associations for AC allele (C/T) was traced back to parent K5; ASV alleles (G/A, A/G) were located in parents ART2-4L3P1-2-1, BG400-1, JARIBU and SUPA TZ; while the co-localised QTLs for GW and L/W came from JARIBU, BR4 and ART3-8L6P3-2-2-B. These parents are potential sources of major effect QTLs for grain quality traits that can be exploited for rice crop improvement. In addition, the results of this study suggested that genetic progress can be attained by intercrossing genotypes from TZ with MDG and CIAT which appeared to be distantly related. Furthermore, from this study, we identified useful targets for QTL validation, fine mapping and cloning that will help rice breeders in contributing to enhancement of rice grain quality traits through marker assisted breeding.

## Supporting information

S1 File Coding of DArTseq SNP data. (XLS)
