# Correction: Diversity analysis and genome-wide association studies of grain shape and eating quality traits in rice (*Oryza sativa* L.) using DArT markers

**DOI:** 10.1371/journal.pone.0212078

**Published:** 2019-02-04

**Authors:** Maurice Mogga, Julia Sibiya, Hussein Shimelis, Daniel Mbogo, Tawanda Muzhingi, Jimmy Lamo, Nasser Yao

Mr. Daniel Mbogo and Dr. Tawanda Muzhingi should be included in the author byline.

Mr. Mbogo should be listed as the fourth author and their affiliation is International Potato Center, CIP, Nairobi, Kenya. The contributions of this author are as follows: data curation, methodology, supervision.

Dr. Muzhingi should be listed as the fifth author and their affiliation is International Potato Center, CIP, Nairobi, Kenya. The contributions of this author are as follows: conceptualization, data curation, methodology, supervision.

The correct citation is: Mogga M, Sibiya J, Shimelis H, Mbogo D, Muzhingi T, Lamo J, et al. (2018) Diversity analysis and genome-wide association studies of grain shape and eating quality traits in rice (Oryza sativa L.) using DArT markers. PLoS ONE 13(6): e0198012. https://doi.org/10.1371/journal.pone.0198012

There are errors in the Author Contributions section. The correct contributions are: Conceptualization: MM, TM, NY. Data curation: MM, DM. Formal analysis: MM. Funding acquisition: NY. Investigation: MM. Methodology: MM, DM, TM. Software: NY. Supervision: JS, HS, DM, TM, JL, NY. Validation: MM. Writing–original draft: MM. Writing–review & editing: MM, JS, HS, DM, TM, JL, NY.

There is information missing from the Funding section. The correct statement is: The work presented here was led by Maurice Mogga and funded by Biosciences eastern and central Africa (BecA), Nairobi, Kenya (ABC 149); Alliance for a Green Revolution in Africa (AGRA PASS 060), Nairobi, Kenya; International Foundation for Science (IFS), Sweden (C_5828_1). This work was partially supported by the BecA-ILRI Hub through the Africa Biosciences Challenge Fund (ABCF) program. The ABCF Program is funded by the Australian Department for Foreign Affairs and Trade (DFAT) through the BecA-CSIRO partnership; the Syngenta Foundation for Sustainable Agriculture (SFSA); the Bill & Melinda Gates Foundation (BMGF); the UK Department for International Development (DFID) and the Swedish International Development Cooperation Agency (Sida). The funders had no role in study design, data collection and analysis, decision to publish, or preparation of the manuscript.

The following information is missing from the Acknowledgments section: Njunge Vincent and Machuka Eunice of BecA-ILRI Hub for their contribution in data generation, collection and analysis.
